# Case Report: Oxaliplatin-Induced Third-Degree Atrioventricular Block: First Discovery of an Important Side-Effect

**DOI:** 10.3389/fcvm.2022.900406

**Published:** 2022-06-27

**Authors:** Xi Chen, Hui Wang, Zijin Zhang, Yan Xu, Xuanqi An, Xin Ai, Lin Li

**Affiliations:** ^1^Department of Medical Oncology, Beijing Hospital, National Center of Gerontology, Institute of Geriatric Medicine, Chinese Academy of Medical Sciences, Beijing, China; ^2^Department of Cardiology, Fuwai Hospital, National Center for Cardiovascular Diseases, Chinese Academy of Medical Sciences, Beijing, China; ^3^Department of Medical Oncology, National Cancer Center/National Clinical Research Center for Cancer/Cancer Hospital, Chinese Academy of Medical Sciences and Peking Union Medical College, Beijing, China

**Keywords:** oxaliplatin, adverse event, cardiotoxicity, third-degree atrioventricular block, coronary artery spasm

## Abstract

**Background:**

The adverse effects of anticancer therapy in patients with malignancies and cardiovascular diseases are complicated. Oxaliplatin is one of the most commonly used chemotherapy drugs for gastric and colorectal cancers, and oxaliplatin-induced cardiotoxicity has rarely been reported.

**Case Summary:**

We report a 76-year-old man with adenocarcinoma of the esophagogastric junction and a 40-day history of non-ST-elevation myocardial infarction who exhibited a new third-degree atrioventricular block after oxaliplatin administration. We immediately withdrew oxaliplatin treatment and, to avoid future episodes, we implanted a permanent pacemaker for safety and added diltiazem hydrochloride. The third-degree atrioventricular block disappeared after oxaliplatin withdrawal. We detected no recurrence of the third-degree atrioventricular block in future chemotherapies.

**Conclusions:**

This is the first reported oxaliplatin-induced third-degree atrioventricular block, likely mediated by coronary artery spasm. Cancer patients with acute coronary syndrome are a unique and vulnerable population, whom physicians should carefully evaluate and monitor during anticancer treatment. Remarkably, even the most common chemotherapy drugs can cause life-threatening cardiac adverse events.

## Introduction

Malignancies and cardiovascular diseases have the highest mortality (1). With treatment development, the prognosis of patients with malignancies and cardiovascular diseases has dramatically improved. However, the adverse effects of anticancer therapy in such patients are complicated, due to their comorbidities.

Oxaliplatin is widely used in treating gastric and colorectal cancers, and effectively prevent DNA replication by forming platinum-DNA adducts, stopping the cell cycle, and ultimately leading to mitotic cell apoptosis. Neurotoxicity is its most unique adverse reaction (2), while cardiotoxicity has rarely been reported.

## Case Presentation

A 76-year-old man visited our outpatient clinic complaining of abdominal discomfort with no history of drug allergy. Gastroscopy detected an ulcerated 2-cm diameter mass in the cardia, lying within 1.5 cm of the dentate line. The pathology revealed moderately to poorly differentiated adenocarcinoma, partly mucinous adenocarcinoma, with a mixed type in Laurén's classification. Chest and abdominal enhanced computed tomography suggested possible lymph node metastasis of the hepatogastric ligament without distant metastasis. According to the Union for International Cancer Control/American Joint Committee on Cancer tumor–node–metastasis staging system (8th Edition, 2017), the clinical stage was stage IIIB (T3N2M0).

**Figure 1:**
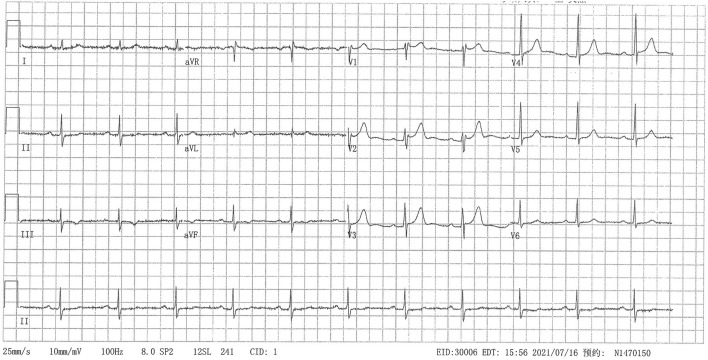
Twelve-lead electrocardiogram showed lead II, III, aVF ST-segment depression of 0.1 mv with T-wave inversion.

Hospitalized in the surgical department, the patient suddenly developed precordial pain with heavy sweat. The electrocardiogram (ECG) suggested dynamic lead II, III, aVF ST-segment depression of 0.1 mv with T-wave inversion (Figure 1). The cardiac troponin I peaked at 0.8299 ng/ml (0–0.0175) the next day. Echocardiography detected moderate (grade II) left ventricular diastolic dysfunction and preserved left ventricular systolic function without regional wall motion abnormalities. The diagnosis of non-ST-elevation myocardial infarction was suspected. Physicians administered anticoagulants and dual antiplatelet and lipid-lowering therapies. Coronary angiography detected 25% stenosis in the mid-left anterior descending artery, 50% stenosis in the first diagonal artery, 75% stenosis in the second diagonal artery, 50% stenosis in the left circumflex artery, 25% stenosis in the middle right coronary artery, 25–50% stenosis in the distal right coronary artery, and 50% stenosis of the post lateral artery. No stent was placed. He had previously received medications for secondary prevention of coronary heart disease, including nitrates. Postoperative ECG and cardiac enzymes returned to normal. The surgeons did not consider the patient a candidate for surgery or radiotherapy due to the higher risks, and they referred him to our medical oncology department for further anti-tumor treatments.

**Figure 2:**
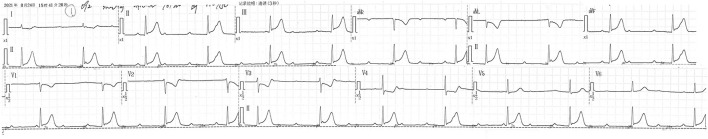
The electrocardiogram identified a third-degree atrioventricular block.

**Figure 3:**
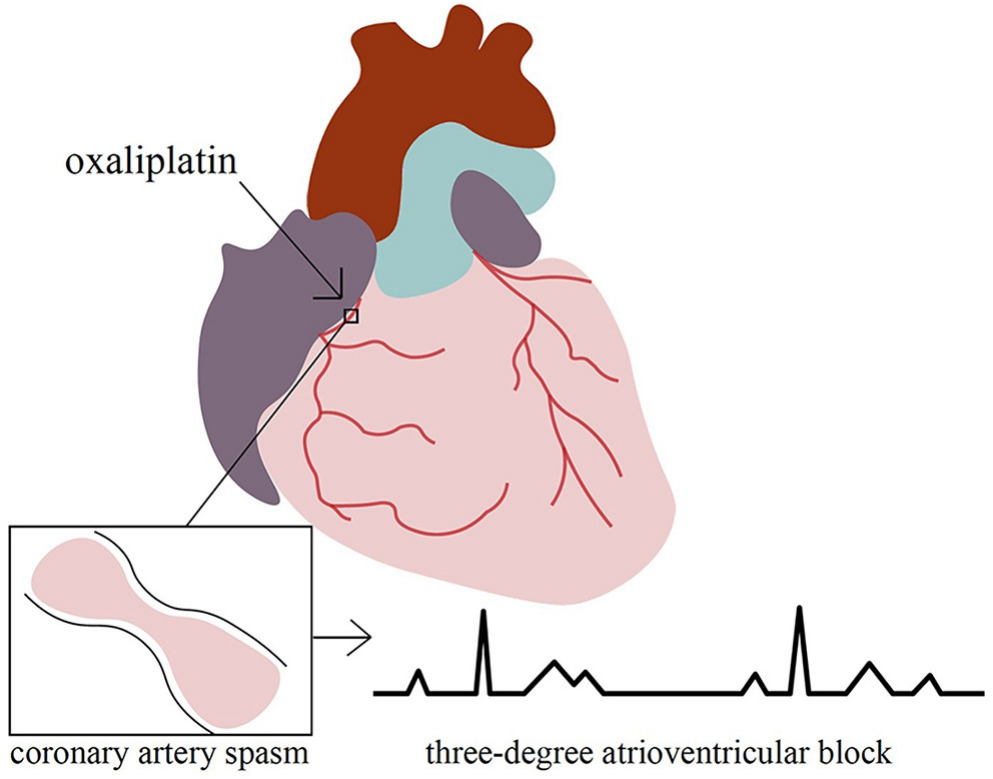
We present a unique case of oxaliplatin-induced third-degree atrioventricular block mediated by coronary artery spasms.

Upon admission to the medical oncology department, the patient reported no chest discomfort with a negative troponin level. He had a temperature of 36.4°C, pulse rate of 72 bpm, breathing rate of 17 breaths per minute, and blood pressure of 125/71 mmHg. Physical examination was unremarkable. We implemented a chemotherapy course consisting of oxaliplatin and tegafur/gimeracil/oteracil and he received oxaliplatin (150 mg) infusion over 1 h. After 1 h of infusion, the patient suddenly complained of shortness of breath, palpitation, and diaphoresis. The patient was alert and oriented. Physical examination revealed a heart rate of 37 bpm, breathing rate of 26 breaths per minute, and blood pressure of 110/50 mmHg. The patient was afebrile. His face was pale and no rash was seen. Cardiovascular examination was notable for a bradycardic rhythm. The ECG identified a new third-degree atrioventricular block (Figure 2). At that time, the patient did not receive tegafur/gimeracil/oteracil. We immediately withdrew oxaliplatin, and the symptoms resolved spontaneously after 20 mins, with the ECG restoring to baseline. His cardiac troponin I cardiac enzymes peaked at 0.0643 ng/ml, 9 h after the onset. Repeated echocardiography revealed no significant changes from baseline. We suspected coronary spasm triggered by oxaliplatin as the etiology, and we administered diltiazem to prevent future onsets. A permanent pacemaker was implanted for safety. The patient complained of no discomfort during the subsequent courses of oxaliplatin and tegafur/gimeracil/oteracil. The pacemaker had not been activated by a low heart rate, and we did not find a third-degree atrioventricular block when we turned down the pacemaker rate during follow-up. A detailed timeline for the case and a graphical abstract image are provided (Table 1, Figure 3).

**Table 1:** Timeline for the case.

**Timeline**	
7 July 2021	Diagnosis of non-ST-segment elevation myocardial infarction
17 July 2021	Asymptomatic with negative troponin level
23 Aug 2021, 14:30	Administration of oxaliplatin over 1 h
23 Aug 2021, 15:38	Developed shortness of breath, palpitation, and diaphoresis.
23 Aug 2021, 15:41	Electrocardiogram detected third-degree atrioventricular block. Decided to withhold tegafur/gimeracil/oteracil
23 Aug 2021, 17:22	Transferred to the cardiology department of our hospital
25 Aug 2021, 02:09	Troponin level peaked
26 Aug 2021	Administration of diltiazem. Pacemaker implantation

## Discussion

In the present case, a 76-year-old patient with adenocarcinoma of the esophagogastric junction underwent cancer therapy and suffered from acute non-ST segment elevation myocardial infarction before surgery. Because he was unable to undergo surgery or radiotherapy, we implemented tailored chemotherapy with oxaliplatin and tegafur/gimeracil/oteracil. Unexpectedly, he developed a third-degree atrioventricular block during oxaliplatin infusion, with newly elevated troponin levels. Having suspected coronary spasms triggered by oxaliplatin, we administered diltiazem to prevent future onsets and implanted a permanent pacemaker. The patient reported no discomfort during future chemotherapies, and the pacemaker programming detected no third-degree atrioventricular blocks.

Anti-cancer therapies, particularly chemotherapy, can account for increased risks of cardiovascular disease and mortalities (3). However, in cases of sudden arrhythmia, it is difficult to determine whether arrhythmias are secondary to chemotherapy or the baseline cardiovascular disease, because baseline clinical data are often lacking (4).

We used the Naranjo adverse drug reaction probability scale (5) to evaluate the relationship between chemotherapeutic drugs and arrhythmia in this patient. We calculated the sum of scores, ranging from – 4 to + 13, to evaluate whether a causal relationship existed. Our patient received his first-time 1-h intravenous infusion of oxaliplatin and developed his first third-degree atrioventricular block at the end of the infusion, which suggested that the arrhythmia was temporally related to the use of oxaliplatin. Additionally, the third-degree atrioventricular block could not be explained by concomitant medications, including dexamethasone and tropisetron hydrochloride. Therefore, we calculated the sum of scores as 5, which meant that oxaliplatin likely caused the arrhythmia.

Oxaliplatin is a third-generation platinum-based drug, and it is one of the most commonly used chemotherapy drugs for gastric and colorectal cancers (6). The adverse reactions of oxaliplatin mainly include gastrointestinal, blood and peripheral nervous system disorders, hypersensitivity reactions, and even anaphylactic shock (7). However, there are few clinical reports of oxaliplatin-induced cardiotoxicity. Oxaliplatin is usually provided without cardiac monitoring, which may lead to the underreporting of the cardiotoxicity caused by it, especially when patients are asymptomatic. Furthermore, there have been no literature reports on oxaliplatin-induced third-degree atrioventricular blocks.

Regarding the possible pathophysiology of our case, because prior coronary angiography ruled out the possibility of acute coronary occlusion, the most likely etiology of the spontaneously resolved third-degree atrioventricular block and mildly elevated troponin was coronary artery spasm. Coronary artery spasm, also known as Primzmetal angina or variant angina, was first described in 1959 (8). Its mechanism is assumed to be an increase in coronary artery's vascular smooth muscle tone. rather than blockage caused by plaque or thrombus. Complications of coronary artery spasm include myocardial necrosis, syncope, arrhythmia (such as ventricular tachycardia and third-degree atrioventricular block), and even cardiac arrest. Certain chemotherapeutics, especially 5-fluorouracil (9), have been documented to cause myocardial ischemia by inducing coronary arterial spasms. We found a total of three cases of oxaliplatin-induced coronary spasms. One patient with colorectal cancer metastasizing to the liver received an oxaliplatin infusion (170 mg) and developed T inversion of lead I, II, aVF, and V3 to V6 on the ECG (10). His coronary angiogram was unremarkable. Two other patients with normal coronary angiograms mimicked ST-elevation myocardial infarction in the leads II, III, and aVF (11, 12). In our patient, the third-degree atrioventricular block was accompanied by a narrow QRS complex, suggesting an atrioventricular nodal block or His bundle block.

A possible pathophysiology of oxaliplatin-induced coronary spasms may be through hyperexcitable voltage-gated Na+ channels (10, 13); another may be via inflammatory mediators such as histamine or leukotriene released during allergic injury, triggered by oxaliplatin-induced degranulation of mast cells and basophils after IgE binding (14–16). One article depicted a case of oxaliplatin-induced type 1 Kounis syndrome (11), a simultaneous occurrence of acute coronary events and hypersensitivity allergic reactions, manifesting as coronary artery spasms. One study found that oxaliplatin-free intervals and premedication with dexamethasone and antihistamines could affect the occurrence of oxaliplatin-related allergic reactions (17). Our patient was treated with dexamethasone before chemotherapy, and he exhibited no signs of allergies, such as skin rash, chest tightness, breathlessness, or anaphylactic shock during the infusion. Therefore, we excluded the diagnosis of Kounis syndrome.

Treatment of coronary artery spasms includes avoiding predisposing factors (such as smoking or vasoactive drugs) and long-acting calcium channel blockers or nitrates. However, their interventions, such as balloon angioplasty, coronary artery bypass surgery, or drug-eluting coronary stent implantation, are ineffective (18). Therefore, we prescribed the patient diltiazem and continued the original chemotherapy at the patient's request. He remained asymptomatic during the subsequent three-course chemotherapy and was satisfied with the overall treatment. At follow-up, the pacemaker had not been activated by a low heart rate, and we did not find a third-degree atrioventricular block when we turned down its rate.

## Conclusions

To the best of our knowledge, this is the first reported case of an oxaliplatin-induced third-degree atrioventricular block. Our patient provided us the opportunity to understand that cancer patients with acute coronary syndrome comprise a unique and vulnerable population who often receive suboptimal anti-cancer and cardiovascular treatment. Therefore, the baseline cardiac status of these patients must be thoroughly evaluated before chemotherapy to avoid unnecessary iatrogenic harm. Moreover, monitoring during treatment and close follow-ups are necessary to ensure immediate and long-term safety. Finally, the management experience for this population was limited to observational studies, as patients with acute coronary syndrome are often excluded from randomized controlled trials. Therefore, we urgently need more clinical experience and research data to assist when formulating treatment plans. However, there are some limitations to this article. The patient refused a cardiac magnetic resonance imaging and further electrophysiological examination or coronary angiography to further confirm the cause, due to financial reasons. This oxaliplatin adverse event needs to be further explored in future work.

## Data Availability Statement

The original contributions presented in the study are included in the article/supplementary material, further inquiries can be directed to the corresponding author/s.

## Ethics Statement

Ethical review and approval was not required for the study on human participants in accordance with the local legislation and institutional requirements. The patients/participants provided their written informed consent to participate in this study.

## Author Contributions

Conceptualization: XC, HW, ZZ, and XAn. Data curation: XC, YX, and XAi. Writing—original draft: XC. Writing—review and editing: LL. All authors contributed to the article and approved the submitted version.

## Funding

CAMS Innovation Fund for Medical Sciences (2021-12M-1-012) financed this study.

## Conflict of Interest

The authors declare that the research was conducted in the absence of any commercial or financial relationships that could be construed as a potential conflict of interest.

## Publisher's Note

All claims expressed in this article are solely those of the authors and do not necessarily represent those of their affiliated organizations, or those of the publisher, the editors and the reviewers. Any product that may be evaluated in this article, or claim that may be made by its manufacturer, is not guaranteed or endorsed by the publisher.

## References

[1] Heron M. Deaths: leading causes for 2018. Natl Vital Stat Rep. (2021) 70:1–115. 34029179

[2] Kang L Tian Y Xu S Chen H. Oxaliplatin-induced peripheral neuropathy: clinical features, mechanisms, prevention, and treatment. J Neurol. (2021) 268:3269–82. 10.1007/s00415-020-09942-w 32474658

[3] Alvarez-Cardona JA Ray J Carver J Zaha V Cheng R Yang E. Cardio-oncology education and training: JACC council perspectives. J Am Coll Cardiol. (2020) 76:2267–81. 10.1016/j.jacc.2020.08.079 33153587 PMC8174559

[4] Hersh MR Linn W Kuhn JG Von Hoff DD. Electrocardiographic monitoring of patients receiving phase I cancer chemotherapy. Cancer Treat Rep. (1986) 70:349–52. 3955546

[5] Naranjo CA Busto U Sellers EM Sandor P Ruiz I Roberts EA. A method for estimating the probability of adverse drug reactions. Clin Pharmacol Ther. (1981) 30:239–45. 10.1038/clpt.1981.154 7249508

[6] Raymond E Faivre S Woynarowski JM Chaney SG. Oxaliplatin: mechanism of action and antineoplastic activity. Semin Oncol. (1998) 25:4–12. 9609103

[7] Yu Z Huang R Zhao L Wang X Shangguan X Li W. Safety profile of oxaliplatin in 3,687 patients with cancer in china: a post-marketing surveillance study. Front Oncol. (2021) 11:757196. 10.3389/fonc.2021.757196 34745993 PMC8567037

[8] Prinzmetal M Kennamer R Merliss R Wada T Bor N. Angina pectoris. I A variant form of angina pectoris; preliminary report. Am J Med. (1959) 27:375–88. 10.1016/0002-9343(59)90003-8 14434946

[9] Luwaert RJ Descamps O Majois F Chaudron JM Beauduin M. Coronary artery spasm induced by 5-fluorouracil. Eur Heart J. (1991) 12:468–70. 2040332 10.1093/oxfordjournals.eurheartj.a059919

[10] Samol J Waterston A. Oxaliplatin-induced coronary artery spasm: first report of an important side-effect. BMJ Case Rep. (2009) 2009:bcr06.2008.0334. 10.1136/bcr.06.2008.0334 21686854 PMC3028146

[11] Chang PH Hung MJ Yeh KY Yang SY Wang CH. Oxaliplatin-induced coronary vasospasm manifesting as Kounis syndrome: a case report. J Clin Oncol. (2011) 29:e776–8. 10.1200/JCO.2011.36.4265 21969496

[12] Weidner K Behnes M Haas J Rusnak J Fuerner P Kuska M. Oxaliplatin-induced acute ST-segment elevation mimicking myocardial infarction: a case report. Oncol Res Treat. (2018) 41:52–6. 10.1159/000480661 29402853

[13] Krishnan AV Goldstein D Friedlander M Kiernan MC. Oxaliplatin-induced neurotoxicity and the development of neuropathy. Muscle Nerve. (2005) 32:51–60. 10.1002/mus.20340 15880395

[14] Kounis NG Zavras GM. Histamine-induced coronary artery spasm: the concept of allergic angina. Br J Clin Pract. (1991) 45:121–8. 1793697

[15] Kounis NG. Kounis syndrome (allergic angina and allergic myocardial infarction): a natural paradigm? Int J Cardiol. (2006) 110:7–14. 10.1016/j.ijcard.2005.08.007 16249041

[16] Abdelghany M Subedi R Shah S Kozman H. Kounis syndrome: A review article on epidemiology, diagnostic findings, management and complications of allergic acute coronary syndrome. Int J Cardiol. (2017) 232:1–4. 10.1016/j.ijcard.2017.01.124 28153536

[17] Shen Y Li C Liu W Mao W Qian H Wang H. Clinical Analysis of Hypersensitivity Reactions to Oxaliplatin Among Colorectal Cancer Patients. Oncol Res. (2018) 26:801–7. 10.3727/096504017X15139039328978 29295722 PMC7844702

[18] Slavich M Patel RS. Coronary artery spasm: current knowledge and residual uncertainties. Int J Cardiol Heart Vasc. (2016) 10:47–53. 10.1016/j.ijcha.2016.01.003 28616515 PMC5462634

